# A population-based lifestyle intervention to promote healthy weight and physical activity in people with cardiac disease: The PANACHE (Physical Activity, Nutrition And Cardiac HEalth) study protocol

**DOI:** 10.1186/1471-2261-10-17

**Published:** 2010-04-08

**Authors:** Janice Sangster, Susan Furber, Margaret Allman-Farinelli, Marion Haas, Philayrath Phongsavan, Andy Mark, Adrian Bauman

**Affiliations:** 1 Health Promotion Service, South Eastern Sydney and Illawarra Area Health, NSW, Australia; 2 University of New South Wales, Sydney, Australia; 3 University of Sydney, Sydney, Australia; 4 University of Technology Sydney, Sydney, Australia; 5 Heart Foundation, Australia

## Abstract

**Background:**

Maintaining a healthy weight and undertaking regular physical activity are important for the secondary prevention of cardiovascular disease (CVD). However, many people with CVD are overweight and insufficiently active. In addition, in Australia only 20-30% of people requiring cardiac rehabilitation (CR) for CVD actually attend. To improve outcomes of and access to CR the efficacy, effectiveness and cost-effectiveness of alternative approaches to CR need to be established.

This research will determine the efficacy of a telephone-delivered lifestyle intervention, promoting healthy weight and physical activity, in people with CVD in urban and rural settings. The control group will also act as a replication study of a previously proven physical activity intervention, to establish whether those findings can be repeated in different urban and rural locations. The cost-effectiveness and acceptability of the intervention to CR staff and participants will also be determined.

**Methods/Design:**

This study is a randomised controlled trial. People referred for CR at two urban and two rural Australian hospitals will be invited to participate. The intervention (healthy weight) group will participate in four telephone delivered behavioural coaching and goal setting sessions over eight weeks. The coaching sessions will be on weight, nutrition and physical activity and will be supported by written materials, a pedometer and two follow-up booster telephone calls. The control (physical activity) group will participate in a six week intervention previously shown to increase physical activity, consisting of two telephone delivered behavioural coaching and goal setting sessions on physical activity, supported by written materials, a pedometer and two booster phone calls. Data will be collected at baseline, eight weeks and eight months for the intervention group (baseline, six weeks and six months for the control group). The primary outcome is weight change. Secondary outcomes include physical activity, sedentary time and nutrition habits. Costs will be compared with outcomes to determine the relative cost-effectiveness of the healthy weight and physical activity interventions.

**Discussion:**

This study addresses a significant gap in public health practice by providing evidence for the efficacy and cost-effectiveness of a low cost, low contact, high reach intervention promoting healthy weight and physical activity among people with CVD in rural and urban areas in Australia. The replication arm of the study, undertaken by the control group, will demonstrate whether the findings of the previously proven physical activity intervention can be generalised to new settings. This population-based approach could potentially improve access to and outcomes of secondary prevention programs, particularly for rural or disadvantaged communities.

**Trial Registration:**

ACTRN12610000102077

## Background

Cardiovascular disease (CVD) is the leading cause of death in Australia [1] and contributes significantly to health costs [2]. Maintaining a healthy weight and participating in regular physical activity are important for the secondary prevention of CVD [3]. However many people with CVD are overweight and physically inactive [4,5]. Despite the benefits of attending cardiac rehabilitation (CR) [6,7] many people with CVD continue to be insufficiently physically active regardless of their attendance at CR [8], and there is little evidence of weight loss occurring as a result of CR attendance [9,10]. Of additional concern is that 70-80% of those requiring secondary prevention for CVD do not attend CR [11-13] leaving the majority of people with CVD with unmet health needs. People that do not attend CR are likely to need it more as they have higher risk factor profiles, poorer risk factor knowledge [14] and live further from CR services than those who attend [15].

Thus the challenge is to improve health outcomes for the majority of people with CVD who do not attend CR. In response to this challenge, investigating alternative service models for delivering CR programs shows promise [16]. To determine the characteristics of effective CR, Clark et al conducted a meta-regression of secondary prevention programs for people with CVD. They concluded that shorter programs delivered by generalist staff in non-hospital settings were at least as effective in reducing mortality as the conventional longer CR programs delivered by specialists in hospital settings [17]. Home-based CR has also been found to be as effective as centre-based CR held in settings such as hospitals, gymnasiums or community centres [18].

Distance interventions, delivered remotely via print, telephone or internet, are likely to have improved population reach, accessibility, cost-effectiveness and maintenance of gains at follow-up compared to centre-based programs [19]. Systematic reviews of distance interventions for increasing physical activity [19], telephone-based interventions for promoting physical activity and dietary change [20], and interventions using pedometers to increase physical activity [21] provide support for the effectiveness of these types of interventions, however conclusions are limited by the quality of the studies assessed. Combining delivery modes for distance interventions, such as using printed educational materials together with pedometers and telephone support, is likely to be most effective [19,20,22].

Reports of interventions promoting healthy weight and physical activity in people with CVD are scarce. A recent Australian study identified significant benefits of a telephone-delivered, pedometer-based intervention on physical activity levels among people with cardiac disease who attended outpatient CR [23]. Another Australian study reported that coaching using telephone and written materials was effective in reducing the body mass index (BMI) of cardiac patients [24]. For overweight patients attending CR, a high-volume, high energy expenditure physical activity program (60-90 minutes per session five-seven days per week) resulted in significant weight reduction compared to standard CR [25,26].

Even fewer interventions address the majority of cardiac patients who do not attend CR. The CHOICE program included a face-to-face consultation and four follow-up phone calls and was effective in improving cardiac risk factors for people with cardiac disease who had not attended CR [5]. Furber et al found that a pedometer-based telephone intervention increased physical activity levels in cardiac patients who did not attend a CR program [27].

Cost effectiveness analyses are rarely conducted on health care interventions [28] and to our knowledge no cost-effectiveness studies of Australian CR programs have been published. A meta-analysis of 63 secondary prevention programs for people with CVD found that few studies published any data on costs and conclusions about cost-effectiveness could not be made [29]. Information on cost-effectiveness of health interventions is useful before health programs are widely implemented. It is recommended that CR research evaluate economic outcomes alongside clinical outcomes [29].

### Objectives of this research

The PANACHE (Physical Activity, Nutrition And Cardiac HEalth) randomised control trial will investigate if a home-based approach (a telephone-delivered, lifestyle intervention that focuses on healthy weight and high-volume physical activity) can decrease obesity and increase physical inactivity in people with CVD in urban and rural areas in Australia. It will also determine whether the outcomes of an intervention previously found to increase physical activity [13,27] are replicated in the control arm of the present study. The cost-effectiveness of the intervention and its suitability in both rural and urban areas of Australia will also be determined.

## Methods/Design

### Study design

**Figure 1:**
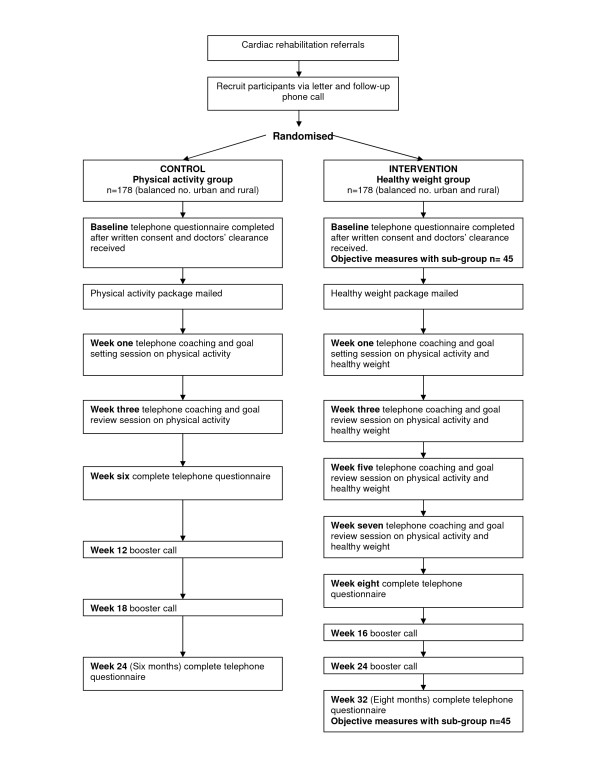
**Design of the PANACHE study**.

This study is a randomised controlled trial comparing the efficacy of a healthy weight telephone coaching intervention (intervention group) with a physical activity telephone coaching intervention (control group). An outline is shown in Figure 1. The control group will also act as a replication study of a telephone-based physical activity intervention previously shown to be efficacious [13,27], and will build on the evidence base by determining whether the findings of the previous study [13,27] can be generalised to diverse settings. An additional advantage of this design is that the attention-focused control group will reduce possible Hawthorne effects (where participants change their performance in response to being observed [30]) for the intervention group in this study.

Approval to conduct this research has been granted by the Human Research Ethics Committees from University of Wollongong, South Eastern Sydney and Illawarra Area Health Service, Greater Southern Area Health Service and the University of New South Wales. 

### Participants

All people referred to outpatient CR over a 12-18 month period at two Sydney and two NSW rural hospitals in Australia will be invited to participate in the study, whether or not they attend outpatient CR. Based on a previous Australian study [13], it is anticipated that of those invited to the study 29% will attend outpatient CR, while 71% will not attend CR. Participants will be excluded if they have any of the following: a clinical diagnosis of uncompensated, severe cardiac failure (Class IV); uncontrolled arrhythmia or angina; severe or symptomatic aortic stenosis; persistent hypotension; a clinical diagnosis of a severe coexisting medical condition that would prevent participation (eg. cognitive impairment, dementia, a terminal illness, severe rheumatoid arthritis, severe arthritis, renal disease requiring dialysis, uncontrolled diabetes); major orthopaedic surgery likely to affect mobility planned within the next 6 months (eg. hip replacement; knee reconstruction; spinal surgery); insufficient English to participate in the telephone coaching calls; non-return of a signed participant consent form and/or a signed doctor's clearance form.

Participants will be recruited six-eight weeks after referral to CR by which time they would be likely to be clinically stable in their recovery process. Brief written information about the study will be included in the information packs distributed to people referred to CR. A personalised invitation letter will also be mailed, followed up with a telephone call.

### Randomisation and blinding

During the follow up telephone call participants will be randomised by the researcher into intervention and control groups when they agree to be enrolled in the study. Participants will be block randomized within site. Microsoft Excel will be used to generate random numbers and the Statistical Analysis System (SAS) will be used to randomise these numbers into sets of two letters (A and B, representing the intervention and control groups respectively) by blocks of four to ensure a balanced sample size across both study groups [31]. The random numbers will be generated and packaged by a person external to the study so the group allocation will be concealed from the researcher until the participant has agreed to be in the study [32]. Participants will not be told whether they have been allocated to the intervention or control group and will remain blinded to group allocation. Group allocation will not be concealed from the researcher when delivering the intervention or assessing outcomes.

### Intervention group

The intervention (healthy weight) group will receive an eight week healthy weight intervention based on social cognitive theory [33,34] which will focus on increasing participants self-efficacy (beliefs about the positive health consequences of taking action) and their use of planning strategies for healthy eating and regular physical activity. It comprises four behavioural coaching and goal setting sessions on weight, nutrition and high-volume physical activity via telephone; and written materials, lifestyle calendar and a pedometer via mail. Participants will be taught how to self-monitor their food intake and physical activity and to use this information to set attainable nutrition and physical activity goals. Goal attainment will be reviewed at subsequent sessions and participants will be assisted to develop strategies to overcome barriers encountered. The first telephone session will take approximately 30 minutes and subsequent calls 10 to 15 minutes depending on the support required. The telephone coaching sessions will be implemented using written telephone coaching guides.

Goals will be individualized and if the participant's BMI is greater than 24.9 kg/m^2 ^participants will be recommended to lose weight [3] and to undertake 60-90 minutes of physical activity on most days. If the participant's BMI is in the healthy weight range of18.5-24.9 kg/m^2 ^the focus will be on weight maintenance [3] and 30 minutes of physical activity on most days of the week will be recommended. Participants will receive two booster phone calls after the intervention to offer feedback on goal attainment and support.

The healthy weight intervention was piloted with nine rural participants. The findings were used to refine evaluation questions and procedures, modify the telephone coaching guide and improve the suitability of written support materials.

### Control group

The control (physical activity) group will receive the same six week physical activity intervention previously found to be efficacious [13,27]. Also based on social cognitive theory, it includes a pedometer and step recording calendar via mail and two behavioural coaching and goal setting sessions (on physical activity, and recommending 30 minutes of physical activity on most days of the week) via telephone as well as two booster phone calls after the intervention.

### Data collection

Questionnaires will be completed by telephone at baseline, eight weeks and eight months for the intervention group and at baseline, six weeks and six months for the control group. Researchers administering the questionnaires will be trained to follow written standard procedures. They will be supervised during the administration of the initial questionnaires and thereafter at random intervals. All objective measures will be obtained by the same researcher following a written standard protocol.

### Process evaluation measures

Semi-structured interviews will be conducted with urban and rural CR staff to assess their views of the usefulness and acceptability of the program and its implementation in their setting. Focus groups will be held with rural and urban study participants regarding their experiences of the program and its delivery. Participants will also provide information on process measures when they complete the telephone questionnaires at week eight (intervention) and week six (control) on the acceptability of program activities and materials such as resources on weight control, nutritional and physical activity, and coaching advice. The weight, nutrition and physical activity goals set by participants during their telephone coaching sessions will also be recorded.

### Outcome measures

The primary outcome is self-reported weight and BMI [3]. Secondary outcomes include self-reported physical activity, sedentary time and nutrition habits. The Active Australia Questionnaire [35], which has demonstrated validity in Australian community [36] and clinical populations [23,37], will be used to assess self-reported total physical activity per week. Sedentary time will be assessed using the question on usual week day sitting time from the International Physical Activity Questionnaire which also has demonstrated reliability and validity [38]. Nutrition habits will be assessed using questions on food intake from the NSW Population Health Survey [39]. Confidence, planning intentions and social support for healthy eating and physical activity will be assessed using questions adapted from previous studies [13,40-42]. Quality of life will be measured using the Assessment of Quality of Life (AQoL) questionnaire which measures quality of life in the domains of independent living, social relationships, physical senses and psychological wellbeing [43]. Quality Adjusted Life Years (QALYs) will also be calculated using the AQoL. The AQoL has been validated for telephone delivery [44] and uses utility weights derived from an Australian population [45].

To validate self-reported changes in height, weight, food intake and physical activity, objective data will be collected at baseline and at eight months from a sub-group of 25% of the intervention group (a convenience sample of 45 participants). At baseline and at eight months the researcher will meet with this sub-group to measure their height, weight and waist circumference after they have self-reported these measures in the questionnaires administered via telephone at baseline and eight months. Participants will then be asked to record their intake of food and drink for three days (two weekdays and one weekend day) and to wear an MTI Actigraph accelerometer to record their physical activity for the next seven days. At the end of the week in which the accelerometer is worn the self-report questions on physical activity, sedentary activity and nutrition habits, which ask about these activities over the last seven days, will be completed. Thus the objective data obtained using a food diary and accelerometer will be collected over the same time period as the self-reported data. A three-day food diary collected over two week days and one weekend day has been found to be a reliable measure of usual energy intake [46]. Accelerometry is a widely accepted method for measuring total movement and provides objective data on the frequency, intensity and duration of physical activity [47].

Costs calculated will include program costs (for example staff time, equipment and telephone costs), direct health care costs related to participants' cardiac conditions (for example emergency department visits, hospital admissions, day procedures, general practitioner and cardiac specialist visits) and other costs (for example participant's expenditure on exercise related products and services such as shoes and exercise classes). Information will be collected regarding the number of days absent from work or normal activities due to cardiac problems.

### Sample size

To detect a reduction of 1.3 kg in weight and 0.5 kg/m^2 ^BMI between the intervention and control group (based on the COACH study effects on weight loss [24]) with a power of 90% (alpha 0.01), adjusting for clustering, and assuming a 20% loss to follow up, a sample size of 178 in each group is required.

### Statistical analysis

The analyses of the trial will be based on (i) intention to treat and (ii) treatment received. Bivariate and multivariate analyses will assess the effects of the intervention (compared to controls) on weight loss, sedentary behaviour, nutrition and physical activity adjusted for residence (rural or urban), age and sex for all cases and then treatment received after initial intention to treat analysis. Continuous data will be analysed with paired t-tests and linear regression and categorical variables with chi square tests and logistic regression with p < 0.05 as the level of significance but adjusted appropriately when multiple testing is conducted. Analyses will be performed with PASW 18.0 (SPSS Inc., Chicago, IL). Food diaries will be analysed using FoodWorks 2007 (Xyris Software).

### Economic analysis

The economic evaluation will be conducted using accepted guidelines [48]. To determine the cost-effectiveness of the intervention, incremental cost-effectiveness ratios will be calculated for any statistically significant outcomes. For the cost-utility analysis, utility values will be calculated using the AQoL questionnaire. If the incremental gain in utility values is statistically significant the incremental cost per QALY will be calculated. The trial results will be used to build a model of future costs and effects beyond the study period. This will be done by extrapolating the intermediate clinical (weight and physical activity levels) and quality of life (QALYs) endpoints to final outcomes (death) using decision modelling based on information from published studies.

### Qualitative analysis

Thematic analysis will be used to examine transcripts of the interviews with CR staff and the focus groups with study participants. Two researchers will independently code the themes arising and then compare and discuss their coding. For the purpose of triangulation, these researchers will then discuss the themes with an additional researcher. The steps taken in the thematic analysis and the reasons for taking them will be documented to provide an audit trail.

## Discussion

Despite the effectiveness of conventional centre-based CR programs, participation rates are low and the majority of people requiring CR are missing out on evidence-based health benefits of lifestyle interventions for cardiac patients. In addition, little research has been conducted on improving health outcomes for the majority of cardiac patients who do not attend CR. This study addresses these gaps in public health practice, firstly by testing an alternative delivery mode for CR, secondly by targeting the entire population of people referred for CR, irrespective of whether they attend a CR program or not, and thirdly by establishing the efficacy of a healthy weight intervention for people with cardiac disease. The economic impact of secondary prevention programs for CVD is an under-researched area. The economic analysis conducted alongside this study will provide important information on the relative costs and benefits of the intervention.

This study will show whether the population-based, low contact, high reach intervention tested can promote healthy weight and physical activity among people with CVD in rural and urban settings and whether it can be delivered cost effectively. By replicating the previously proven physical activity only program [13,27] in the control arm, our study will also demonstrate whether this approach is effective in a range of 'real-life' urban and rural settings.

The findings of this study will have significant implications for the management of people with CVD. In addition to improving health outcomes for people with cardiac disease, these interventions have the potential to reduce costs and improve access to CR services, particularly for disadvantaged and rural people. They could be a feasible addition to existing services and could also be delivered to people with CVD who have already attended CR programs as a "maintenance" program.

## Competing interests

The authors declare that they have no competing interests.

## Authors' contributions

JS drafted the manuscript, contributed to the study design and is coordinating the study. SF, MA-F, MH, PP, AM and AB contributed to the study design, advised on coordination of the study and reviewed the manuscript. All authors read and agreed to the manuscript as written.

## Pre-publication history

The pre-publication history for this paper can be accessed here:

http://www.biomedcentral.com/1471-2261/10/17/prepub
